# Chensinin-1b Alleviates DSS-Induced Inflammatory Bowel Disease by Inducing Macrophage Switching from the M1 to the M2 Phenotype

**DOI:** 10.3390/biomedicines12020345

**Published:** 2024-02-01

**Authors:** Yue Sun, Huiyu Li, Xingpeng Duan, Xiaoxiao Ma, Chenxi Liu, Dejing Shang

**Affiliations:** 1 School of Life Science, Liaoning Normal University, Dalian 116081, China; sunyue01@lnnu.edu.com (Y.S.);; 2 Liaoning Provincial Key Laboratory of Biotechnology and Drug Discovery, Liaoning Normal University, Dalian 116081, China

**Keywords:** antimicrobial peptide, macrophage polarization, inflammatory bowel disease, NF-κB

## Abstract

Inflammatory bowel disease (IBD) is a chronic relapsing inflammatory disorder with an increasing prevalence worldwide. Macrophage polarization is involved in the pathogenesis of IBD. Repolarization of macrophage has thus emerged as a novel therapeutic approach for managing IBD. Chensinin-1b, derived from the skin of *Rana chensinensis*, is a derivative of a native antimicrobial peptide (AMP). It shows anti-inflammatory effects in sepsis models and can potentially modulate macrophage polarization. The objective of this research was to study the role of chensinin-1b in macrophage polarization and dextran sulfate sodium (DSS)-induced colitis. RAW264.7 macrophages were polarized to the M1 phenotype using lipopolysaccharide (LPS) and simultaneously administered chensinin-1b at various concentrations. The ability of chenisnin-1b to reorient macrophage polarization was assessed by ELISA, qRT-PCR, and flow cytometry analysis. The addition of chensinin-1b significantly restrained the expression of M1-associated proinflammatory cytokines and surface markers, including TNF-α, IL-6, NO, and CD86, and exaggerated the expression of M2-associated anti-inflammatory cytokines and surface markers, including IL-10, TGF-β1, *Arg-1*, *Fizz1*, *Chil3*, and CD206. Mechanistically, via Western Blotting, we revealed that chensinin-1b induces macrophage polarization from the M1 to the M2 phenotype by inhibiting the phosphorylation of nuclear factor-kappa B (NF-κB) and mitogen-activated protein kinase (MAPK). In mouse models of colitis, intraperitoneal administration of chensinin-1b alleviated symptoms induced by DSS, including weight loss, elevated disease activity index (DAI) scores, colon shortening, colonic tissue damage, and splenomegaly. Consistent with our in vitro data, chensinin-1b induced significant decreases in the expression of M1 phenotype biomarkers and increases in the expression of M2 phenotype biomarkers in the mouse colitis model. Furthermore, chensinin-1b treatment repressesed NF-κB phosphorylation in vivo. Overall, our data showed that chensinin-1b attenuates IBD by repolarizing macrophages from the M1 to the M2 phenotype, suggesting its potential as a therapeutic candidate for IBD.

## 1. Introduction

Inflammatory bowel disease (IBD) is an idiopathic immune-mediated inflammatory disease that encompasses two primary forms: ulcerative colitis (UC) and Crohn’s disease (CD) [1,2]. IBD is characterized by chronic, progressive, and relapsing inflammatory disorders of the alimentary canal, with symptoms of chronic abdominal pain, persistent diarrhea, maldigestion, weight loss, rectal bleeding, or fatigue [3]. In addition, there is a heightened risk of developing intestinal and extraintestinal malignancies in IBD patients [4,5,6]. IBD is increasing in incidence worldwide and has become a disease of global concern due to urbanization and changes in nutrition and diet; thus, it represents a costly burden for the healthcare system. The pharmacological agents currently used for IBD include antibiotics, aminosalicylates, steroids, immunosuppressants, and anti-tumor necrosis factor (TNF)-α antibody. Despite the effectiveness of the medications, they have certain limitations and side effects that impact their administration. Hence, there is still an unmet need for novel therapeutic options for IBD. Although the etiology of IBD is multifactorial, it ultimately presents to be a chronic inflammation caused by disturbances in immune homeostasis, under the dynamic interactions among genetic, environmental, and developmental factors [7,8,9,10,11]. The failure of the immune system to transition from a proinflammatory toward an anti-inflammatory response hinders the resolution of inflammation. As a result, prolonged inflammation occurs in the intestinal tract, and various immune cells are involved [12,13].

Specifically, macrophages are pivotal modulators of immune and inflammatory response. In addition to a population of self-maintaining embryo-derived macrophages in the intestine, there are bone marrow-derived infiltrating macrophages responsible for replenishing and maintaining intestinal macrophages, even in the absence of infection or inflammation [14]. Together, intestinal-resident macrophages act as key regulators of local intestinal homeostasis, playing multidimensional roles in neuroprotection, vascular protection, immune surveillance, pathogen clearance, tissue repair, the modulation of inflammation in physiological and pathophysiological contexts. The phenotypic shift that occurs in macrophages is associated with the pathogenesis of IBD [15,16,17,18,19]. Macrophages exhibit phenotypic plasticity, which enables them to alter their function as a result of diverse microenvironmental cues via differentiation into M1 (proinflammatory) and M2 (anti-inflammatory) subsets [20,21,22]. In microenvironments dominated by lipopolysaccharide (LPS), either individually or in conjunction with cytokines associated with T-helper (Th) 1 response, such as interferon-γ (IFNγ), M1 macrophage polarization is triggered [23], which activates Th1 immunity and result in substantial production of proinflammatory cytokines to support functions in immune surveillance [24,25]. However, the abundant release of proinflammatory cytokines and mediators evokes exacerbation of inflammation and tissue injury [26,27]. Conversely, M2 macrophages develop in an environment containing Th2-associated cytokines, like interleukin (IL)-4 and IL-13. As opposed to the M1 phenotype, M2 macrophages express anti-inflammatory cytokines to quell inflammation, repair damaged tissue, and facilitate wound healing [28,29].

The dysregulation of macrophage polarization has been associated with the initiation and development of IBD [17,19]. A large amount of evidence demonstrates that proinflammatory M1 macrophages are predominant and promote mucosal barrier lesions and IBD progression in IBD patients, while the M2 phenotype is less abundant [30]. Furthermore, other investigations have suggested that insufficient M2 polarization of macrophages is likely to increase IBD susceptibility and severity [17,31,32,33]. Therefore, regulatory strategies aimed at achieving a fine-tuned equilibrium between M1 and M2 macrophages hold promising potential for the treatment of IBD.

Antimicrobial peptides (AMPs) belong to an important class of small protective molecules, which are cationic, amphipathic, and hydrophobic, constituting an essential composition of innate immunity in various species [34,35,36,37]. AMPs are different from conventional antibiotics and possess a unique membrane-targeting antibacterial mechanism that renders them less prone to the development of bacterial resistance [38,39]. Beyond the well-described antimicrobial activity, AMPs also have been reported to have immunomodulatory activity, including inhibiting inflammation in recent years [40,41,42,43]. Some AMPs have inflammatory suppressing effects by participating in nuclear factor-kappa B (NF-κB), mitogen-activated protein kinase (MAPK), and other signaling cascades [44,45,46]. Furthermore, multiple studies have demonstrated that some AMPs play a regulatory role in macrophage polarization [47,48,49,50]. Chensinin-1 (SAVGRHGRRFGLRKHRKH), derived from the skin secretions of Rana chensinensis, a species of Chinese brown frog, demonstrates moderate antimicrobial effects but no hemolytic activity [51,52]. Additionally, it was reported to interact with LPS and suppress cytokine release in an LPS-stimulated RAW 264.7 macrophage inflammatory model [53]. In a previous study, we synthesized a novel analog, chensinin-1b (amino acid sequence: SKVWRHWRRFWHRAHRLH, molecular weight: 2543.18 Da), through the rearrangement and replacement of amino acids, resulting in enhanced amphiphilicity and hydrophobicity. The chemical structure of chensinin-1b is shown in Figure S1. In comparison to the original template peptide, this modified chensinin-1b has much greater and broader antibacterial activity and stronger antagonistic activity against LPS. In addition, it demonstrates no apparent haemolytic activity (HC50 > 500 mM). Immunomodulatory and anti-inflammatory properties of chensinin-1b have also been reported. The effectiveness of chensinin-1b in promoting wound healing was demonstrated in a murine model of infected wounds [54]. When administered in an experimental murine model of septic shock, chensinin-1b significantly suppressed systemic inflammatory cytokines, attenuated lung and liver tissue damage, and increased the survival of mice [55]. In addition, Dong et al. conjugated chensinin-1b to aliphatic acid, and the new derivative decreased LPS-induced cytokine production from human U937 cells [56]. These findings imply that chensinin-1b has therapeutic potential in the management of inflammatory and immune disorders. Nevertheless, the effect of chensinin-1b on IBD remains unknown. Herein, our objective in this study was to probe the effect of chensinin-1b on IBD, its role in modulating macrophage polarization, and the underlying mechanism. Through in vivo and in vitro experiments, we provided evidence that chensinin-1b impedes macrophage M1 polarization and skews M2 polarization, thus having anti-inflammatory effects on intestinal inflammation in a murine model of DSS-induced colitis.

## 2. Materials and Methods

### 2.1. Peptide Synthesis and Purification

Chensinin-1b was synthesized as per the standard Fmoc solid-phase peptide synthetic approach by GL Biochem Co., Ltd. (Shanghai, China). Reverse-phase HPLC was used to purify the synthesized peptide to 95% homogeneity by using a 2.2 × 25 cm Vydac 218TP1022 C-18 column (Separations Group, Hesperis, CA, USA) equilibrated with acetonitrile/water/tri-fluoroacetic acid. After purification, a MALDI-TOF mass spectrometer (Shimadzu, Kyoto, Japan) was utilized for the detection of the relative mass of chensinin-1b.

### 2.2. Cell Culture and Treatment

The RAW 264.7 murine macrophage line procured from the cell bank of the Chinese Academy of Sciences Type Culture Collection Committee (Shanghai, China) was cultured in DMEM (Gibco, Grand Island, NY, USA) which contained 10% FBS (Gibco, Grand Island, NY, USA), 100 U/mL penicillin, and 100 mg/mL streptomycin. The cells were incubated at a controlled temperature of 37 °C and 5% CO_2_ concentration. These cells were plated in 96-well culture dishes, with 1 × 10^5^ cells/well, to attach overnight. Afterward, Ml polarization was induced through incubation with 1 ug/mL LPS. During the induction of polarization, chensinin-1b was also introduced into the culture at various concentrations (10, 20, and 40 μM) and maintained for 24 h to prepare cells for future experimental analysis. For the isolation of peritoneal macrophages, an anesthetized mouse was given an intraperitoneal injection of 5 mL of cold, sterile PBS. Following a mild abdominal massage to facilitate fluid distribution, the peritoneal lavage fluid was collected and centrifuged. Then, 10% FBS/DMEM was used to resuspend the harvested cells. Peritoneal macrophages were then purified through a process of plastic adherence.

### 2.3. Cell Viability Assay

Proliferation and viability were quantitatively measured using a cell counting kit-8 (CCK-8) assay. Initially, RAW 264.7 cells (2000 cells/well) were plated in 96-well dishes in triplicate and incubated overnight. Subsequently, a series of chensinin-1b concentrations (0, 10, 20, 40, 80, and 160 μM) were administered. After 24 h of incubation at 37 °C, each well received 10 μL of CCK8 reagent (Dojindo, Shanghai, China) and was incubated for an additional 4 h in darkness. The absorbance was recorded at 450 nm utilizing the Varioskan Flash fluorescence spectrophotometer (Thermo Scientific, Waltham, MA, USA). Cell viability ratios were calculated based on the following formula: (optical density of the chensinin-1b treated group/optical density of the blank control group) × 100%.

### 2.4. Measurement of Cytokines by Enzyme-Linked Immunosorbent Assay (ELISA)

The concentrations of cytokines, including TNF-α, IL-6, IL-10, and TGF-β1, in RAW 264.7 cell supernatants or serum of mice were quantified following the guidelines provided by the manufacturer of ELISA kits (Neobioscience Technology, Shanghai, China).

### 2.5. Quantification of Nitric Oxide (NO) Production

To measure the concentration of nitrite within the culture medium, Griess reagent (Beyotime Biotechnology, Shanghai, China) was used to quantify NO production by RAW 264.7 cells as described previously [57]. At room temperature, a 50 µL sample from every group was blended with equivalent volumes of Griess Reagent I and II for 30 min. The absorbance at a wavelength of 540 nm was determined using a spectrophotometer (Varioskan Flash, Thermo Scientific, Waltham, MA, USA). In every experiment, a fresh culture medium was invariably applied as a blank control.

### 2.6. Western Blotting

Total proteins from cells and mouse tissues were extracted with RIPA lysis buffer (Thermo Scientific, Waltham, MA, USA) supplemented with a cocktail of protease and phosphatase inhibitors (MedChemExpress, Monmouth Junction, NJ, USA). To quantify the concentration of these proteins, a QubitTM Protein BR Assay Kit (Thermo Scientific, Waltham, MA, USA) was employed. Equivalent quantities of sample proteins were denatured using a loading buffer. The proteins were then transferred to PVDF membranes (Millipore, Beijing, China) after being resolved via 10% SDS–polyacrylamide gel electrophoresis (SDS–PAGE). After 2 h incubation in blocking solution (5% skimmed milk in TBST), the membrane was rinsed with TBST and subsequently incubated in blocking solution overnight at 4 °C with primary antibodies against various proteins, such as NF-κB p65, phosphorylated NF-κB p65, IκBα, phosphorylated IκBα, ERK, phosphorylated ERK, JNK, phosphorylated JNK, p38, phosphorylated p38, and GAPDH (all at a dilution of 1:1000, except phosphorylated ERK and GAPDH, with a dilution of 1:2000), sourced from Cell Signaling Technology (Shanghai, China). Detection involved the application of HRP-conjugated goat anti-rabbit IgG from Abcam, Cambridge, UK (diluted 1:2000) for 1 h at ambient temperature. The visualization of protein bands was facilitated using enhanced chemiluminescence (ECL) reagent (Tanon, Shanghai, China) and the Azure C500 Gel Imaging System (Azure Biosystems, Dublin, CA, USA). Band analysis and quantification were conducted using ImageJ (v1.43, NIH, Bethesda, MD, USA), with GAPDH as the internal standard for normalization.

### 2.7. Quantitative Real-Time PCR

The extraction of total RNA from RAW 264.7 cells and colon tissues was performed using a TRIzol reagent (Vazyme, Nanjing, China). Subsequently, RNA was reverse-transcribed into cDNA by employing the HiScript^®^ II Q RT SuperMix for qPCR (+gDNA wiper) kit (Vazyme, Nanjing, China), as per the guidelines provided by the manufacturer. qRT-PCR was performed in an ABI-Prism 7500 Fast sequence detection system (Applied Biosystems, Foster City, CA, USA) using TB Green ^®^ Premix Ex Taq™ II (TaKaRa, Dalian, China). The PCR consisted of 40 cycles at 95 °C for 5 s, 60 °C for 30 s, and 70 °C for 30 s. Gene expression levels were analyzed using the 2−ΔΔCT method using the LightCycler^®^ 96 software (Roche Diagnostics, Mannheim, Germany). GAPDH was used for normalization. The primer sequence is provided in Supplementary Table S1.

### 2.8. Animals

SPF BALB/C mice (female, 6–8 weeks, 19–21 g) were obtained from the Liaoning Changsheng Biotechnology company (Shenyang, China). Mice were maintained in animal facilities with a 12:12 h light/dark schedule at a temperature of 20–22 °C and relative humidity of 50–60% and supplied with water and a standard rodent diet ad libitum. Every effort was undertaken to minimize animal usage and suffering.

### 2.9. DSS-Induced Colitis and Treatment

After 4 days of acclimation, the mice were divided into 5 experimental groups (each comprising 5 individuals) in a random manner: normal control, colitis control, cyclosporin A (CsA)-treated (25 mg/kg), low-dose chensinin-1b-treated (1.5 mg/kg), and high-dose chensinin-1b-treated (3 mg/kg). The mice in the normal control group received plain drinking water, contrasting with the other groups, which received DSS-containing water (4%, *w*/*v*) for a duration of 7 days to induce acute colitis. The treated mice received daily intraperitoneal injections of CsA or chensinin-1b from day 1 to day 9, whereas the control group was injected with saline. Based on established protocols, the disease activity index (DAI) was calculated daily, consisting of assessments of body weight fluctuations and stool consistency as well as instances of rectal bleeding [58]. On day 9, mice were humanely euthanized via cervical dislocation under isoflurane to collect serum, peritoneal macrophages, the colon, and the spleen for further studies. Colon length and spleen weight were recorded.

### 2.10. Flow Cytometry

RAW 264.7 cells or peritoneal macrophages were harvested and rinsed with 2% BSA/PBS. Following the cleansing process, the cells were stained for 30 min at a temperature of 37 °C. The staining involved the use of PE-conjugated anti-mouse CD86 or CD206. To establish a baseline for comparison, PE-conjugated rat IgG2a, κ (Biolegend, San Diego, CA, USA) was employed as an isotype control. Stained cells were analyzed using a BD FACSVerseTM flow cytometer (BD Biosciences, Franklin Lakes, NJ, USA), and fluorescence intensities were determined using the FlowJoTM v10.0.7 software.

### 2.11. H&E Staining and Histology

Colon tissue samples were preserved in 4% paraformaldehyde solution, and 5 µm sections were prepared using paraffin-embedded samples. Sections were then subjected to a deparaffinization process using xylene, followed by a graded ethanol series for dehydration. After being rinsed with tap water, the sections were stained in a sequential manner using hematoxylin and eosin. The Olympus CKX41 microscope (Tokyo, Japan) was utilized to examine the pathological alterations within the colon tissues. Histological scoring methods were adopted from a previous report [59]. Histological assessments were performed blindly to ensure an unbiased evaluation.

### 2.12. Immunohistochemistry Analysis

The expression levels and distribution of CD86, CD206, and NF-κB p65 in colon tissue were visualized via immunohistochemical analysis. After the dewaxing rehydration step as mentioned above, sections were probed overnight at 4 °C with the primary antibodies, targeting CD86, CD206, and phospho-NF-κB p65 (all at a dilution of 1:1000) sourced from Cell Signaling Technology. The second antibody (ZSGB-BIO, Beijing, China) was applied and incubated at 37 °C. The Olympus CKX41 microscope (Tokyo, Japan) was utilized for photography.

### 2.13. Statistical Analysis

Data are presented as mean ± SEM, calculated from three independent experimental runs. For each of these runs, the analysis of individual samples was conducted in triplicate to ensure consistency and accuracy. The statistical evaluation of the data was performed using the Origin 2021 software from Origin Lab. The differences between various experimental groups were determined through statistical tests, including the Student’s *t*-test and one-way ANOVA, supplemented by Tukey’s multiple comparison test when needed. A *p*-value threshold of less than 0.05 was established for considering the results as statistically significant.

## 3. Results

### 3.1. The Effect of Chensinin-1b on the Expression of Biomarkers Associated with M1 and M2 Macrophage Polarization

To study the regulatory function of chensinin-1b on macrophage polarization, this study employed a commonly utilized RAW 264.7 murine macrophage cell line. We first tested the viability of RAW264.7 cells after exposure to chensinin-1b using the CCK8 assay to determine an appropriate concentration for this research. The results revealed that chensinin-1b had no significant cytotoxicity to RAW 264.7 cells, with more than 80% of cells surviving at concentrations below 40 μM (Figure S2).

**Figure 1:**
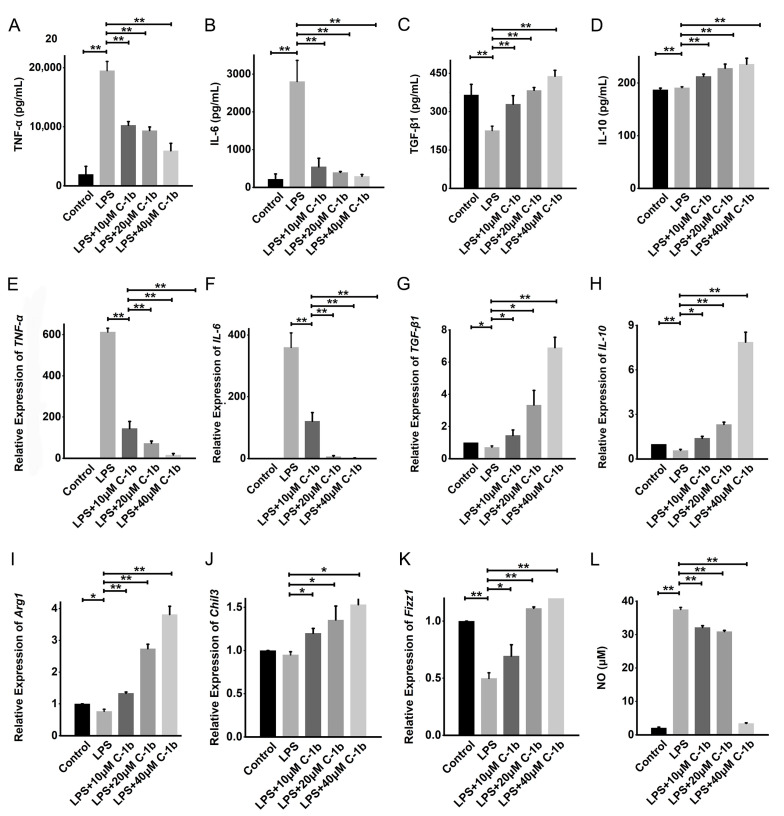
Chensinin-1b decreased proinflammatory cytokine levels and increased anti-inflammatory cytokine levels in LPS-stimulated RAW 264.7 cells. Cells were cultured with chensinin-1b (10, 20, or 40 μM) in the presence of LPS (1 μg/mL) for 24 h. The levels of proinflammatory (TNF-α and IL-6) and anti-inflammatory (IL-10 and TGF-β1) cytokines were assessed using ELISA (**A**–**D**). The mRNA expression of TNF-α, IL-6, IL-10, TGF-β1, Arg-1, Chil3, and Fizz-1 was quantified using qRT-PCR analysis (**E**–**K**). NO content was measured using the Griess reagent (**L**). Data represent the mean ± SEM (*n* = 3), with * *p* < 0.05 and ** *p* < 0.01.

Therefore, concentrations lower than the IC20 of chensinin-1b were selected and used for subsequent experiments. We constructed a model of macrophage M1 polarization via LPS treatment of RAW 264.7 cells to analyze the impact of chensinin-1b on M1-polarized macrophages. The levels of M1/M2-associated cytokines and genes were detected in LPS-activated macrophages treated with or without chensinin-1b by ELISA, Griess assays, and RT-PCR (Figure 1). In comparison to the control group, LPS stimulation increased the production of NO and the expression of the proinflammatory cytokines TNF-α and IL-1β. Chensinin-1b treatment suppressed the expression of M1-associated cytokines and mediators stimulated by LPS in a dose-dependent manner.

**Figure 2:**
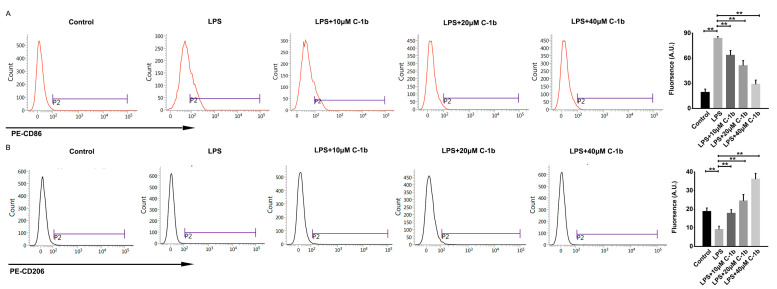
Chensinin-1b altered M1 polarization in LPS-stimulated RAW 264.7 cells. Cells were treated with chensinin-1b (0, 10, 20, or 40 μM) in the presence of 1 μg/mL LPS for 24 h. The expression levels of CD86 (**A**) and CD206 (**B**) were examined by flow cytometry. Data represent the mean ± SEM (*n* = 3), with ** *p* < 0.01.

Conversely, LPS decreased the expression of M2-associated cytokines and genes (TGF-β1, IL-10, Arg-1, Chil3, and Fizz1), but this effect was reduced by chensinin-1b treatment (Figure 1B). Furthermore, flow cytometry analysis revealed decreased expression of CD86 (an M1-type surface maker) but increased the expression of the M2-type surface maker CD206 in M1-polarized macrophages subjected to chensinin-1b treatment (Figure 2). Collectively, these data implied that chensinin-1b may skew M1-polarized macrophages induced by LPS towards an M2-dominant phenotype.

### 3.2. The Effect of Chensinin-1b on the Modulation of the NF-κB and MAPK Pathways in LPS-Stimulated RAW 264.7 Cells

**Figure 3:**
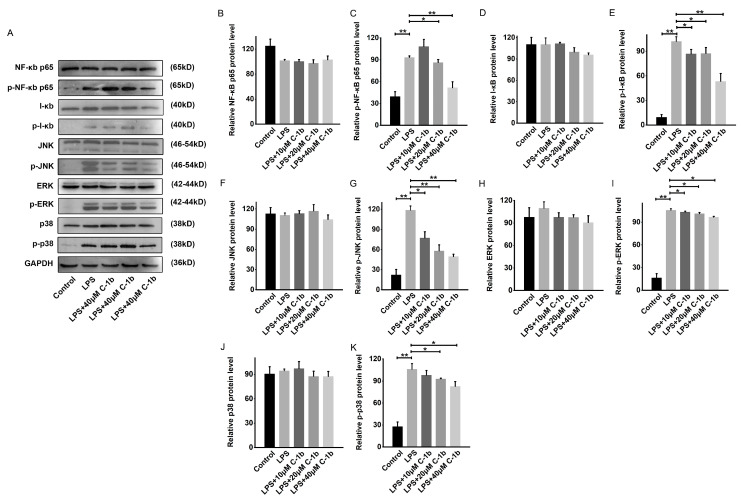
Chensinin-1b suppressed the NF-κB and MAPK pathways in LPS-stimulated RAW 264.7 cells (**A**–**K**). Cells were subjected to treatment with chensinin-1b (0, 10, 20, or 40 μM) with the presence of 1 μg/mL LPS for 24 h. Western Blot analysis was performed to assess the protein expression of NF-κB p65, phospho-NF-κB p65, I-κB, phospho-I-κB, JNK, phospho-JNK, ERK, phospho-ERK, p38, and phospho-p38. Data represent the mean ± SEM (*n* = 3), with * *p* < 0.05 and ** *p* < 0.01.

The NF-κB and MAPK signaling pathways are critically involved in initiating the proinflammatory response and promoting M1 macrophage polarization [60]. Since chensinin-1b skews macrophage polarization in LPS-stimulated RAW 264.7 cells, we explored whether chensinin-1b affects the levels of phosphorylated and total NF-κB p65, I-κB, JNK, ERK, and p38 via Western Blotting. According to the findings depicted in Figure 3, the phosphorylation of these proteins was augmented following LPS stimulation, illustrating the activation of the NF-κB/MAPK pathway in the context of M1 macrophage polarization in RAW 264.7 cells. However, chensinin-1b treatment resulted in the dose-dependent inhibition of these phosphorylation events. These results demonstrate that chensinin-1b suppresses M1 polarization and facilitates M2 polarization by repressing the NF-κB/MAPK pathway.

### 3.3. Chensinin-1b Ameliorated DSS-Induced Colitis in Mice

**Figure 4:**
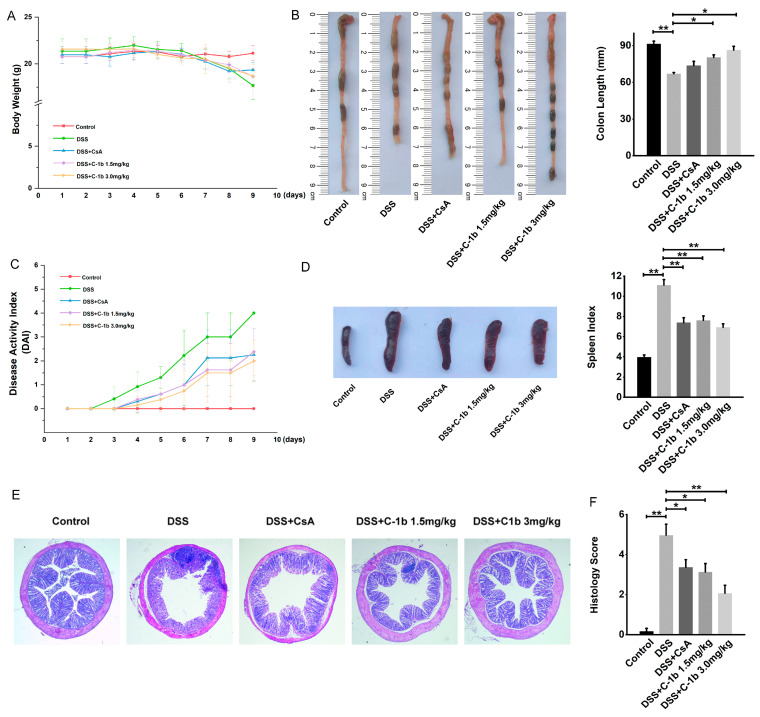
Chensinin-1b ameliorated the severity of DSS-induced colitis in mice. BALB/C mice received drinking water containing 4% DSS for 7 days and were simultaneously treated with chensinin-1b (1.5 or 3 mg/kg intraperitoneally) daily from day 1 to day 9. Body weight changes (**A**) and DAI scores (**C**) during DSS-induced colitis were recorded daily. Mice were euthanized on day 9. The distal colon and spleen were gathered to assess colon length (**B**) and spleen index (**D**). Colonic tissue damage was identified through H&E staining (**E**) and was quantified as histological scores (**F**). Data represent the mean ± SEM (*n* = 5 mice per group), with * *p* < 0.05 and ** *p* < 0.01.

The pivotal role of macrophages in the pathogenesis of IBD has been well-documented [15,17,19,61]. An imbalance in the M1/M2 macrophage ratio was related to colitis progression in murine models of IBD [44,45]. In mice with experimental DSS-induced colitis, an increase in the M1 macrophage population coupled with a decrease in M2 macrophages has been noted. Thus, the repression of M1 macrophages and the mobilization of M2 macrophages might represent novel therapeutic strategies for IBD. Since chensinin-1b could correct the bias toward the M1 phenotype in LPS-treated macrophages, we investigated whether chensinin-1b has a beneficial effect on IBD. To this end, a DSS-induced colitis model was established by administering 4% DSS in drinking water for 9 days, and chensinin-1b was given via peritoneal injection. Mice administered DSS developed severe colitis, which contributed to weight loss, as opposed to the control group with normal drinking water. In contrast, chensinin-1b treatment markedly mitigated body weight loss (Figure 4A). Consistent with this result, scores for the DAI, commonly utilized as an indicator for assessing colitis severity based on parameters like diarrhea and rectal bleeding, exhibited a significant decrease in chensinin-1b-treated mice compared to saline-treated ones (Figure 4C). The physical manifestations of colitis, such as colonic shortening and spleen enlargement, were evident in DSS-treated mice, with a notable decrease in colon length and an increase in spleen index (spleen weight/body weight). Chensinin-1b administration led to an improvement in colon length (Figure 4B) and a reduction in spleen index (Figure 4D). Histological examinations also revealed that chensinin-1b ameliorated colon submucosal edema, infiltration of inflammatory cells, and crypt architecture disruption in colitis mice (Figure 4E), with a significant decline in histology scores observed in the chensinin-1b-treated group compared to the colitis group (Figure 4F). These findings collectively indicate that chensinin-1b treatment can alleviate symptoms in mice with DSS-induced colitis, with high doses group exhibiting stronger therapeutic effects than CsA positive control, potentially due to the regulatory impact on macrophage phenotypic polarization.

### 3.4. Chensinin-1b Alters Macrophage Polarization in Mice with DSS-Induced Colitis

**Figure 5:**
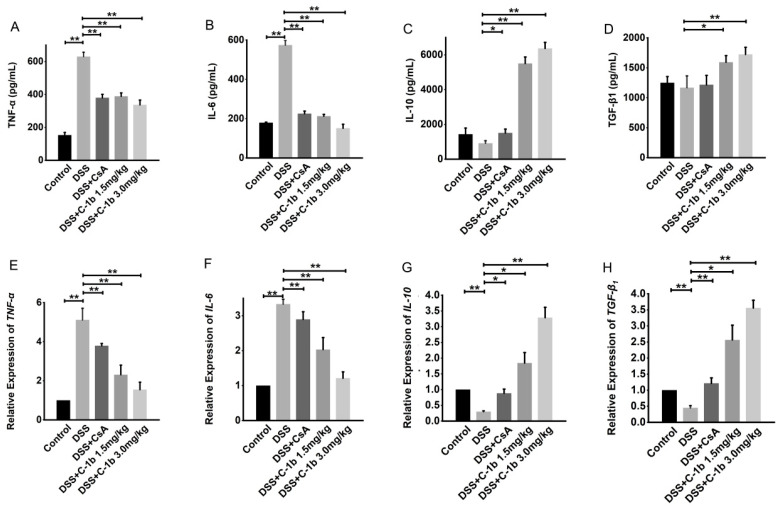
The treatment of chensinin-1b regulated the expression of pro- and anti-inflammatory cytokines in mice with DSS-induced colitis. Serum levels of the cytokines TNF-α, IL-6, IL-10, and TGF-β1 (**A**–**D**) are shown. The mRNA expression of cytokines, including TNF-α, IL-6, IL-10, and TGF-β1, in colon tissue (**E**–**H**) is shown. Data represent the mean ± SEM (*n* = 5 mice per group), with * *p* < 0.05 and ** *p* < 0.01.

To further determine the potential therapeutic efficacy of chensinin-1b on DSS-induced colitis and its association with the shift in macrophage phenotypes, this study assessed the cytokine profiles in serum and colon tissues. Specifically, the levels of TNF-α, IL-6, IL-10, and TGF-β1 were measured using ELISA and qRT-PCR. As illustrated in Figure 5, there was a noticeable reduction in the expression of pro-inflammatory cytokines TNF-α and IL-6, while anti-inflammatory cytokines IL-10 and TGF-β1 showed an increase after chensinin-1b treatment, both in serum and colon tissues. This modulation of cytokine levels occurred in a dose-dependent manner and was statistically significant (*p* < 0.05), indicating the potential of chensinin-1b in rebalancing the cytokine milieu toward a more anti-inflammatory state in the context of IBD.

**Figure 6:**
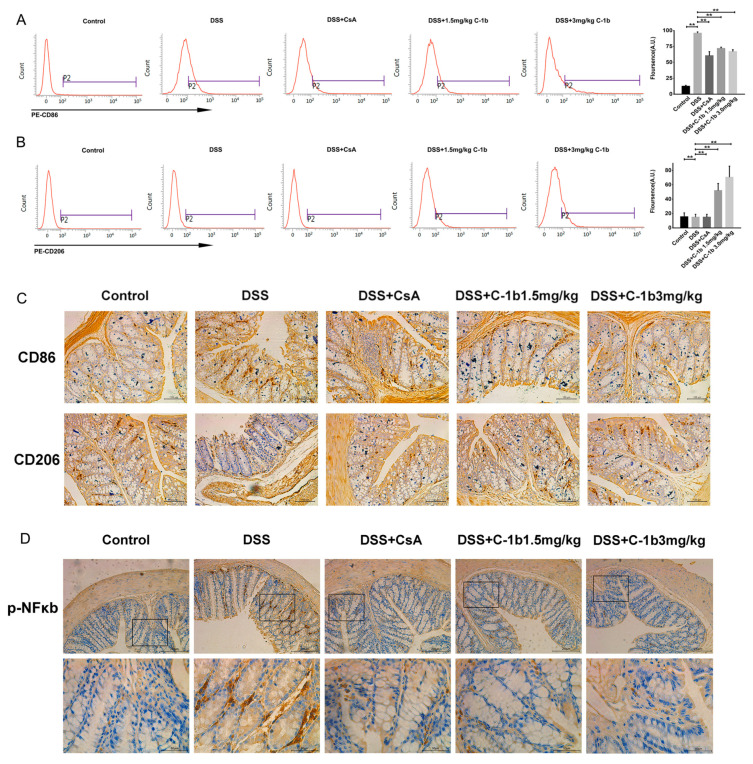
Chensinin-1b modulated macrophage polarization in mice with DSS-induced colitis. The expression of CD86 (**A**) and CD206 (**B**) in peritoneal macrophages was detected using flow cytometry. Immunohistochemical staining was performed to analyze the expression of CD86 and CD206 in colon tissue. Scale bars represent 100 μm (**C**). The immunohistochemical analysis of phospho-NF-κB p65 in colon tissue (**D**) is shown. Boxed regions in the upper row (scale bars, 200 µm) are shown magnified below (scale bars, 50 µm). Data represent the mean ± SEM (*n* = 5 mice per group), with ** *p* < 0.01.

In addition to cytokine profiling, we also evaluated surface markers indicative of M1 and M2 macrophage phenotypes in peritoneal macrophages. Flow cytometry analysis, as depicted in Figure 6A,B, showed that, in colitis mice, peritoneal macrophages predominantly expressed CD86 with relatively lower levels of CD206. Post-treatment with chensinin-1b led to a significant reduction in CD86 expression, while there was a corresponding increase in CD206 expression. Similar results were obtained for colon tissues, as evidenced in Figure 6C. Immunohistochemical staining of colon tissues revealed a marked decrease in CD86-positive macrophages and an increase in CD206-positive macrophages in mice treated with chensinin-1b, compared to those with colitis. Furthermore, the expression of NF-κB p65 in colon tissues, visualized through immunohistochemical analysis (Figure 6D), was substantially elevated in mice with colitis compared to healthy controls. However, similar to the in vitro findings, chensinin-1b treatment mitigated this increase, consistent with the observed phenotypic shifts in macrophages. Altogether, these data provide evidence that chensinin-1b reduces the severity of DSS-induced colitis in mice. This reduction is largely due to its ability to repolarize M1 macrophages to M2 phenotype, thereby effectively addressing the inflammation. These insights affirm the potential of chensinin-1b as a therapeutic approach in IBD management, emphasizing its crucial role in modulating macrophage polarization and inflammatory processes.

## 4. Discussion

IBD is a disease with high incidence worldwide, and there is no effective therapy to completely arrest the progression of IBD. The pathogenesis of IBD involves overzealous intestinal inflammation coupled with dysregulated innate immune homeostasis. AMPs have been attracting attention due to their anti-inflammatory and immunomodulatory properties. Chensinin-1b is a synthetic derivative of native AMP, originally isolated from *Rana chensinensis*. However, whether chensinin-1b has a therapeutic effect on IBD remains unclear. Herein, we demonstrate for the first time the efficacy of chensinin-1b in an experimental IBD animal model by balancing M1/M2 macrophage polarization to attenuate inflammation.

Macrophages are highly adaptable cells that undergo an M1/M2 phenotypic switch in response to microenvironmental stimuli and signals [62]. Macrophages are essential elements of innate immunity, and disordered macrophage polarization has been shown to be involved in the initiation and progression of many inflammatory disorders, such as IBD. In IBD patients and colitis animal models, monocytes/macrophages accumulate in the intestines. They overreact to TLR ligands and produce too many cytokines and chemokines, preventing inflammation resolution [63]. Numerous studies have shown that shifting macrophage polarization from the proinflammatory M1 phenotype to the anti-inflammatory M2 phenotype helps to relieve intestinal inflammation and restore tissue function [64,65,66]. Thus, repolarization of macrophages is a potential treatment strategy for IBD.

AMPs are small multifunctional molecules that are important for the immune system and have well-known antimicrobial activity. Furthermore, a large amount of evidence suggests that some AMPs are able to regulate macrophage polarization and are thus potentially therapeutic in a variety of inflammatory-mediated disease models. Chensinin-1b is a synthetic derivative of native AMP originally identified from *Rana chensinensis*, with a broader spectrum and enhanced antimicrobial properties [54,55,56]. Additionally, we have previously demonstrated that chensinin-1b improves tissue repair and sepsis outcomes in wound infection and sepsis models [67,68], indicating a potential function in the repolarization of macrophages. Therefore, we focused on the possible role of chensinin-1b in repolarizing macrophages and treating inflammatory diseases. This study is the first to reveal that treatment with chensinin-1b promoted macrophage polarization toward a noninflammatory phenotype and in turn relieved IBD. We demonstrated that chesinin-1b alleviates DSS-induced colitis by reducing M1 macrophages and promoting the accumulation of M2 macrophages. The protective mechanisms of chensinin-1b are associated with the suppression of NF-κB and MAPK signaling cascades. We present evidence that chensinin-1b likely acts as an immune-regulatory peptide in addition to its antimicrobial activities.

Initially, we evaluated chensinin-1b’s effect on macrophage polarization using RAW264.7 cells exposed to LPS to induce the M1 phenotype in vitro. Differential cytokine production is key to the cellular activity of polarized macrophages. Our study reveals that chensinin-1b effectively suppresses the production of M1-associated proinflammatory cytokines while simultaneously enhancing M2 marker expression at a nontoxic dose in vitro.

The NF-κB pathway is a classical proinflammatory signaling cascade that leads to the overproduction of proinflammatory cytokines by macrophages. NF-κB p65 is one of the pivotal mediators that boost M1 macrophage polarization [62,69,70]. Upon stimulation by LPS, I-κB undergoes phosphorylation and degradation, which triggers the release and nuclear translocation of p50/p65 heterodimer. P65 subsequently alters M1-associated gene expression [71]. Our results confirm that chensinin-1b inhibits the LPS-induced phosphorylation of I-κB and p65. In addition, the MAPK family members, ERK, JNK, and p38, are highly conserved serine/threonine protein kinases in mammals that are implicated in a variety of macrophage functions. Reports have demonstrated that the activation of the MAPK cascade is often associated with M1 macrophage polarization. These pathways can promote the expression of M1 macrophage-associated genes via the phosphorylation and activation of AP-1 transcription factors like c-Jun and c-Fos. Furthermore, evidence has shown that p38 and JNK induce I-κB degradation, which activates the NF-κB p65 pathway [72,73]. Herein, we found that chensinin-1b restricted the activation of MAPK pathways induced by LPS. In addition to chensinin-1b, similar effects of other AMPs in amphibian skin have also been reported. For instance, cathelicidin-PP and Nv-CATH are two cathelicidin-like AMPs isolated from Polypedates puerensis and Nanorana ventripunctata, respectively [74,75]. Brevinin-1FL and revinine-2MP belong to the revinine family of AMPs, which were identified from the skin of *Fejervarya limnocharis* and *Microhyla pulchra* [76,77]. Analogues of temporin-1Cea, temporin-1Ceb, and temporin-1Cec, namely LK2(6), L-K6, and 2K4L, were derived from the skin secretion of *Rana chensinensis* [78,79,80].

All these peptides inhibit both NF-κB and MAPK signaling in macrophages, and our results are consistent with these previous studies, suggesting that suppression of the M1 macrophage proinflammatory phenotype is associated with the NF-κB and MAPK pathways. Increased M1/M2 macrophage ratio is reported in IBD [81,82], and a skew from M1 to M2 macrophages could favor the resolution of intestinal inflammation [83,84]. Considering the pivotal involvement of macrophage polarization in both the development and resolution of IBD, as well as the modulatory properties of chensinin-1b on macrophage polarization by shifting it from an M1 to an M2 phenotype, we examined the efficacy of chensinin-1b in an experimental colitis model. The mouse model of DSS-induced colitis exhibits similarities to human colitis [85] and is appropriate for investigating the role of innate immune cells in intestinal inflammation [86]. Chensinin-1b treatment resulted in a marked amelioration in the symptoms of DSS-induced colitis, including weight loss, DAI score, colon length shortening, spleen swelling, and colonic pathological damage. Simultaneously, the elevated expression of M1 biomarkers and decreased expression of M2 biomarkers were also reverted with chensinin-1b administration. These data showed that the efficacy of chensinin-1b for IBD was attributed to the transition of macrophage polarization from M1 to M2.

Further, proper control of the NF-κB cascade remains an attractive approach for treating IBD due to the hyperactivation of the disease [87,88]. It has been reported that a variety of agents that have efficacy in IBD are known to inhibit the NF-κB pathway [33,89,90,91]. These include some peptide-based drugs, such as Cathelicidin-BF, WFNNAGP, LP-5, pep-1, APE, and LL-37-Tα1 [92,93,94,95,96,97]. Immunohistochemical examination of the colon tissue showed that chensinin-1b effectively suppressed the expression of NF-κB p65. This is consistent with our in vitro results, indicating an association between the anti-inflammatory properties of chensinin-1b and its impact on NF-κB signaling pathways in intestinal inflammation. However, further identification of the exact targets for intervention by chensinin-1b is still needed.

On the other hand, gut microbes could also be implicated in IBD pathogenesis. Both animal and human studies have shown that there are significant alterations in the composition of the gut microbiota between IBD patients and healthy persons, including alterations in microbial diversity and the relative abundance of bacterial taxa [71]. Moreover, the microbiota affects intestinal homeostasis and inflammation by activating TLRs and NOD-like receptors (NLRs) [72]. Antibiotic administration and fecal microbiota transplantation have beneficial effects on colonic inflammation in IBD [73]. Considering that chensinin-1b was demonstrated to be effective against a diverse range of microbiota [54,55,67], in the future, it will be necessary to investigate whether the therapeutic effects of chensinin-1b on colitis are correlated with alterations in the gut microbiota beyond direct regulation of macrophage polarization. Due to the intricate nature of IBD and the beneficial role of M2 macrophages in repairing IBD-associated barrier defects, further investigation is warranted to elucidate the potential of chensinin-1b in IBD intervention, epithelial barrier restoration, and gut microbiota modulation. Moreover, while chensinin-1b exhibits low cytotoxicity in macrophages and demonstrates good tolerance in mice, additional toxicological data will be essential for its progression into clinical trials.

In conclusion, we demonstrated that chensinin-1b reorients macrophage polarization from the M1 to M2 type by altering the NF-κB/MAPK signaling pathways to resolve inflammation and relieve DSS-induced colitis in a mouse model. These results show that chensinin-1b has the potential to be a promising alternative for treating IBD and other associated inflammatory disorders.

## Data Availability

All data that support the findings presented in this paper are available upon request from the corresponding author.

## Supplementary Materials

The following supporting information can be downloaded at: https://www.mdpi.com/article/10.3390/biomedicines12020345/s1, Figure S1: The chemical structure of chensinin-1b; Figure S2: Effect of chensinin-1b on RAW 264.7 cell viability. Cells were administrated with gradient concentrations (0, 10, 20, 40, 80, 160 μM) of chensinin-1b for 24 h. Cell viability was test using CCK-8 assay. Data represent the mean ± SEM, *n* = 3; Table S1: Primers for qPCR.

## References

[1] Danese S., Argollo M., Le Berre C., Peyrin-Biroulet L. (2019). JAK selectivity for inflammatory bowel disease treatment: Does it clinically matter?. Gut.

[2] Friedrich M., Pohin M., Powrie F. (2019). Cytokine Networks in the Pathophysiology of Inflammatory Bowel Disease. Immunity.

[3] Baumgart D.C., Sandborn W.J. (2012). Crohn’s disease. Lancet.

[4] Eaden J.A., Abrams K.R., Mayberry J.F. (2001). The risk of colorectal cancer in ulcerative colitis: A meta-analysis. Gut.

[5] Everhov Å.H., Erichsen R., Sachs M.C., Pedersen L., Halfvarson J., Askling J., Ekbom A., Ludvigsson J.F., Sørensen H.T., Olén O. (2020). Inflammatory bowel disease and pancreatic cancer: A Scandinavian register-based cohort study 1969–2017. Aliment. Pharmacol. Ther..

[6] Axelrad J.E., Olén O., Sachs M.C., Erichsen R., Pedersen L., Halfvarson J., Askling J., Ekbom A., Sørensen H.T., Ludvigsson J.F. (2021). Inflammatory bowel disease and risk of small bowel cancer: A binational population-based cohort study from Denmark and Sweden. Gut.

[7] Zhang Y.Z., Li Y.Y. (2014). Inflammatory bowel disease: Pathogenesis. World J. Gastroenterol..

[8] Lee S.H., Kwon J.E., Cho M.L. (2018). Immunological pathogenesis of inflammatory bowel disease. Intest. Res..

[9] Dixon L.J., Kabi A., Nickerson K.P., McDonald C. (2015). Combinatorial effects of diet and genetics on inflammatory bowel disease pathogenesis. Inflamm. Bowel Dis..

[10] Park J.H., Peyrin-Biroulet L., Eisenhut M., Shin J.I. (2017). IBD immunopathogenesis: A comprehensive review of inflammatory molecules. Autoimmun. Rev..

[11] Ng S.C., Bernstein C.N., Vatn M.H., Lakatos P.L., Loftus E.V., Tysk C., O’Morain C., Moum B., Colombel J.F. (2013). Geographical variability and environmental risk factors in inflammatory bowel disease. Gut.

[12] Neurath M.F. (2014). Cytokines in inflammatory bowel disease. Nat. Rev. Immunol..

[13] De Souza H.S., Fiocchi C. (2016). Immunopathogenesis of IBD: Current state of the art. Nat. Rev. Gastroenterol. Hepatol..

[14] De Schepper S., Verheijden S., Aguilera-Lizarraga J., Viola M.F., Boesmans W., Stakenborg N., Voytyuk I., Schmidt I., Boeckx B., Dierckx de Casterlé I. (2018). Self-Maintaining Gut Macrophages Are Essential for Intestinal Homeostasis. Cell.

[15] Sanchez-Munoz F., Dominguez-Lopez A., Yamamoto-Furusho J.K. (2008). Role of cytokines in inflammatory bowel disease. World J. Gastroenterol..

[16] Zhu Y., Zhou J., Feng Y., Chen L., Zhang L., Yang F., Zha H., Wang X., Han X., Shu C. (2018). Control of Intestinal Inflammation, Colitis-Associated Tumorigenesis, and Macrophage Polarization by Fibrinogen-Like Protein 2. Front. Immunol..

[17] Hunter M.M., Wang A., Parhar K.S., Johnston M.J., Van Rooijen N., Beck P.L., McKay D.M. (2010). In vitro-derived alternatively activated macrophages reduce colonic inflammation in mice. Gastroenterology.

[18] Yang Y.F., Zhou Y.D., Hu J.C., Luo F.L., Xie Y., Shen Y.Y., Bian W.X., Yin Z.N., Li H.L., Zhang X.L. (2017). Ficolin-A/2, acting as a new regulator of macrophage polarization, mediates the inflammatory response in experimental mouse colitis. Immunology.

[19] Li C., Xu M.M., Wang K., Adler A.J., Vella A.T., Zhou B. (2018). Macrophage polarization and meta-inflammation. Transl. Res..

[20] Glass C.K., Natoli G. (2016). Molecular control of activation and priming in macrophages. Nat. Immunol..

[21] Na Y.R., Stakenborg M., Seok S.H., Matteoli G. (2019). Macrophages in intestinal inflammation and resolution: A potential therapeutic target in IBD. Nat. Rev. Gastroenterol. Hepatol..

[22] Mowat A.M., Bain C.C. (2011). Mucosal macrophages in intestinal homeostasis and inflammation. J. Innate Immun..

[23] Mosser D.M., Edwards J.P. (2008). Exploring the full spectrum of macrophage activation. Nat. Rev. Immunol..

[24] Mantovani A., Sica A., Locati M. (2005). Macrophage polarization comes of age. Immunity.

[25] Muraille E., Leo O., Moser M. (2014). Th1/Th2 paradigm extended: Macrophage polarization as an unappreciated patho-gen-driven escape mechanism?. Front. Immunol..

[26] Bain C.C., Mowat A.M. (2014). Macrophages in intestinal homeostasis and inflammation. Immunol. Rev..

[27] Shapouri-Moghaddam A., Mohammadian S., Vazini H., Taghadosi M., Esmaeili S.A., Mardani F., Seifi B., Mohammadi A., Afshari J.T., Sahebkar A. (2018). Macrophage plasticity, polarization, and function in health and disease. J. Cell Physiol..

[28] Murray P.J., Wynn T.A. (2011). Protective and pathogenic functions of macrophage subsets. Nat. Rev. Immunol..

[29] Gordon S., Martinez F.O. (2010). Alternative activation of macrophages: Mechanism and functions. Immunity.

[30] Dharmasiri S., Garrido-Martin E.M., Harris R.J., Bateman A.C., Collins J.E., Cummings J.R.F., Sanchez-Elsner T. (2021). Human Intestinal Macrophages Are Involved in the Pathology of Both Ulcerative Colitis and Crohn Disease. Inflamm. Bowel Dis..

[31] Takada Y., Hisamatsu T., Kamada N., Kitazume M.T., Honda H., Oshima Y., Saito R., Takayama T., Kobayashi T., Chinen H. (2010). Monocyte chemoattractant protein-1 contributes to gut homeostasis and intestinal inflammation by composition of IL-10-producing regulatory macrophage subset. J. Immunol..

[32] Caër C., Wick M.J. (2020). Human intestinal mononuclear phagocytes in health and inflammatory bowel disease. Front. Immunol..

[33] Zhu Y., Li X., Chen J., Chen T., Shi Z., Lei M., Zhang Y., Bai P., Li Y., Fei X. (2016). The pentacyclic triterpene Lupeol switches M1 macrophages to M2 and ameliorates experimental inflammatory bowel disease. Int. Immunopharmacol..

[34] Zasloff M. (2002). Antimicrobial peptides of multicellular organisms. Nature.

[35] Kang H.K., Kim C., Seo C.H., Park Y. (2017). The therapeutic applications of antimicrobial peptides (AMPs): A patent review. J. Microbiol..

[36] Lei J., Sun L., Huang S., Zhu C., Li P., He J., Mackey V., Coy D.H., He Q. (2019). The antimicrobial peptides and their potential clinical applications. Am. J. Transl. Res..

[37] Yan Y., Li Y., Zhang Z., Wang X., Niu Y., Zhang S., Xu W., Ren C. (2021). Advances of peptides for antibacterial applications. Colloids Surf. B Biointerfaces.

[38] Raheem N., Straus S.K. (2019). Mechanisms of Action for Antimicrobial Peptides with Antibacterial and Antibiofilm Functions. Front. Microbiol..

[39] Seyfi R., Kahaki F.A., Ebrahimi T., Montazersaheb S., Eyvazi S., Babaeipour V., Tarhriz V. (2020). Antimicrobial Peptides (AMPs): Roles, Functions and Mechanism of Action. Int. J. Pept. Res. Ther..

[40] Javia A., Amrutiya J., Lalani R., Patel V., Bhatt P., Misra A. (2018). Antimicrobial peptide delivery: An emerging therapeutic for the treatment of burn and wounds. Ther. Deliv..

[41] Pinheiro da Silva F., Machado M.C. (2017). The dual role of cathelicidins in systemic inflammation. Immunol. Lett..

[42] Pizzo E., Cafaro V., Di Donato A., Notomista E. (2018). Cryptic Antimicrobial Peptides: Identification Methods and Current Knowledge of their Immunomodulatory Properties. Curr. Pharm. Des..

[43] Liu C.W., Su B.C., Chen J.Y. (2021). Tilapia Piscidin 4 (TP4) Reprograms M1 Macrophages to M2 Phenotypes in Cell Models of Gardnerella vaginalis-Induced Vaginosis. Front. Immunol..

[44] Mahida Y.R. (2000). The key role of macrophages in the immunopathogenesis of inflammatory bowel disease. Inflamm. Bowel Dis..

[45] Elson C.O., Sartor R.B., Tennyson G.S., Riddell R.H. (1995). Experimental models of inflammatory bowel disease. Gastroenterology.

[46] Krieglstein C.F., Cerwinka W.H., Sprague A.G., Laroux F.S., Grisham M.B., Koteliansky V.E., Senninger N., Granger D.N., de Fougerolles A.R. (2002). Collagen-binding integrin alpha1beta1 regulates intestinal inflammation in experimental colitis. J. Clin. Invest..

[47] Ji S.Y., Lee H., Hwangbo H., Hong S.H., Cha H.J., Park C., Kim D.H., Kim G.Y., Kim S., Kim H.S. (2020). A Novel Peptide Oligomer of Bacitracin Induces M1 Macrophage Polarization by Facilitating Ca(^2+^) Influx. Nutrients.

[48] Bouzazi D., Mami W., Mosbah A., Marrakchi N., Ben Ahmed M., Messadi E. (2023). Natriuretic-like Peptide Lebetin 2 Mediates M2 Macrophage Polarization in LPS-Activated RAW264.7 Cells in an IL-10-Dependent Manner. Toxins.

[49] Ghebremedhin A., Salam A.B., Adu-Addai B., Noonan S., Stratton R., Ahmed M.S.U., Khantwal C., Martin G.R., Lin H., Andrews C. (2020). A Novel CD206 Targeting Peptide Inhibits Bleomycin Induced Pulmonary Fibrosis in Mice. Cells.

[50] Pena O.M., Afacan N., Pistolic J., Chen C., Madera L., Falsafi R., Fjell C.D., Hancock R.E. (2013). Synthetic cationic peptide IDR-1018 modulates human macrophage differentiation. PLoS ONE.

[51] Shang D., Yu F., Li J., Zheng J., Zhang L., Li Y. (2009). Molecular cloning of cDNAs encoding antimicrobial peptide precursors from the skin of the Chinese brown frog, *Rana chensinensis*. Zool. Sci..

[52] Shang D., Sun Y., Wang C., Wei S., Ma L., Sun L. (2012). Membrane interaction and antibacterial properties of chensinin-1, an antimicrobial peptide with atypical structural features from the skin of *Rana chensinensis*. Appl. Microbiol. Biotechnol..

[53] Dong W., Sun Y., Shang D. (2015). Interactions between chensinin-1, a natural antimicrobial peptide derived from Rana chensinensis, and lipopolysaccharide. Biopolymers.

[54] Sun Y., Dong W., Sun L., Ma L., Shang D. (2015). Insights into the membrane interaction mechanism and antibacterial properties of chensinin-1b. Biomaterials.

[55] Shang D., Meng X., Zhang D., Kou Z. (2017). Antibacterial activity of chensinin-1b, a peptide with a random coil conformation, against multiple-drug-resistant *Pseudomonas aeruginosa*. Biochem. Pharmacol..

[56] Dong W., Liu Z., Sun L., Wang C., Guan Y., Mao X., Shang D. (2018). Antimicrobial activity and self-assembly behavior of antimicrobial peptide chensinin-1b with lipophilic alkyl tails. Eur. J. Med. Chem..

[57] Au R.Y., Al-Talib T.K., Au A.Y., Phan P.V., Frondoza C.G. (2007). Avocado soybean unsaponifiables (ASU) suppress TNF-alpha, IL-1beta, COX-2, iNOS gene expression, and prostaglandin E2 and nitric oxide production in articular chondrocytes and monocyte/macrophages. Osteoarthr. Cartil..

[58] Rangan P., Choi I., Wei M., Navarrete G., Guen E., Brandhorst S., Enyati N., Pasia G., Maesincee D., Ocon V. (2019). Fasting-Mimicking Diet Modulates Microbiota and Promotes Intestinal Regeneration to Reduce Inflammatory Bowel Disease Pathology. Cell Rep..

[59] Wirtz S., Neufert C., Weigmann B., Neurath M.F. (2007). Chemically induced mouse models of intestinal inflammation. Nat. Protoc..

[60] Sun Q., Liu Q., Zheng Y., Cao X. (2008). Rapamycin suppresses TLR4-triggered IL-6 and PGE(2) production of colon cancer cells by inhibiting TLR4 expression and NF-kappaB activation. Mol. Immunol..

[61] Kühl A.A., Erben U., Kredel L.I., Siegmund B. (2015). Diversity of Intestinal Macrophages in Inflammatory Bowel Diseases. Front. Immunol..

[62] Sica A., Mantovani A. (2012). Macrophage plasticity and polarization: In vivo veritas. J. Clin. Investig..

[63] Moreira Lopes T.C., Mosser D.M., Gonçalves R. (2020). Macrophage polarization in intestinal inflammation and gut homeostasis. Inflamm. Res..

[64] Zhang Y., Li X., Luo Z., Ma L., Zhu S., Wang Z., Wen J., Cheng S., Gu W., Lian Q. (2020). ECM1 is an essential factor for the determination of M1 macrophage polarization in IBD in response to LPS stimulation. Proc. Natl. Acad. Sci. USA.

[65] Zhou X., Li W., Wang S., Zhang P., Wang Q., Xiao J., Zhang C., Zheng X., Xu X., Xue S. (2019). YAP Aggravates Inflammatory Bowel Disease by Regulating M1/M2 Macrophage Polarization and Gut Microbial Homeostasis. Cell Rep..

[66] Wu Y., Wu B., Zhang Z., Lu H., Fan C., Qi Q., Gao Y., Li H., Feng C., Zuo J. (2020). Heme protects intestinal mucosal barrier in DSS-induced colitis through regulating macrophage polarization in both HO-1-dependent and HO-1-independent way. FASEB J..

[67] Dong W., Dong Z., Mao X., Sun Y., Li F., Shang D. (2016). Structure-activity analysis and biological studies of chensinin-1b analogues. Acta Biomater..

[68] Li Z., Qu W., Zhang D., Sun Y., Shang D. (2023). The antimicrobial peptide chensinin-1b alleviates the inflammatory response by targeting the TLR4/NF-κB signaling pathway and inhibits Pseudomonas aeruginosa infection and LPS-mediated sepsis. Biomed. Pharmacother..

[69] Tugal D., Liao X., Jain M.K. (2013). Transcriptional control of macrophage polarization. Arterioscler. Thromb. Vasc. Biol..

[70] Simon P.S., Sharman S.K., Lu C., Yang D., Paschall A.V., Tulachan S.S., Liu K. (2015). The NF-κB p65 and p50 homodimer cooperate with IRF8 to activate iNOS transcription. BMC Cancer.

[71] Hoesel B., Schmid J.A. (2013). The complexity of NF-κB signaling in inflammation and cancer. Mol. Cancer.

[72] Je J.H., Lee J.Y., Jung K.J., Sung B., Go E.K., Yu B.P., Chung H.Y. (2004). NF-kappaB activation mechanism of 4-hydroxyhexenal via NIK/IKK and p38 MAPK pathway. FEBS Lett..

[73] Yeung Y.T., Aziz F., Guerrero-Castilla A., Arguelles S. (2018). Signaling Pathways in Inflammation and Anti-inflammatory Therapies. Curr. Pharm. Des..

[74] Mu L., Zhou L., Yang J., Zhuang L., Tang J., Liu T., Wu J., Yang H. (2017). The first identified cathelicidin from tree frogs possesses anti-inflammatory and partial LPS neutralization activities. Amino Acids.

[75] Shi J., Wu J., Chen Q., Shen Y., Mi K., Yang H., Mu L. (2022). A Frog-Derived Cathelicidin Peptide with Dual Antimicrobial and Immunomodulatory Activities Effectively Ameliorates *Staphylococcus aureus*-Induced Peritonitis in Mice. ACS Infect. Dis..

[76] Chai J., Liu J., Tian M., Liao H., Wu J., Xie J., Lai S., Mo G., Chen X., Xu X. (2022). Multiple Mechanistic Action of Brevinin-1FL Peptide against Oxidative Stress Effects in an Acute Inflammatory Model of Carrageenan-Induced Damage. Oxid. Med. Cell Longev..

[77] Tian M., Liu J., Chai J., Wu J., Xu X. (2021). Antimicrobial and Anti-inflammatory Effects of a Novel Peptide from the Skin of Frog Microhyla pulchra. Front. Pharmacol..

[78] Yang X.F., Liu X., Yan X.Y., Shang D.J. (2023). Effects of frog skin peptide temporin-1CEa and its analogs on ox-LDL induced macrophage-derived foam cells. Front. Pharmacol..

[79] Dong W., Zhu X., Zhou X., Yang Y., Yan X., Sun L., Shang D. (2018). Potential role of a series of lysine-/leucine-rich antimicrobial peptide in inhibiting lipopolysaccharide-induced inflammation. Biochem. J..

[80] Ji F., Tian G., Shang D., Jiang F. (2023). Antimicrobial peptide 2K4L disrupts the membrane of multidrug-resistant Acinetobacter baumannii and protects mice against sepsis. Front. Microbiol..

[81] Zhu W., Yu J., Nie Y., Shi X., Liu Y., Li F., Zhang X.L. (2014). Disequilibrium of M1 and M2 macrophages correlates with the development of experimental inflammatory bowel diseases. Immunol. Investig..

[82] Lissner D., Schumann M., Batra A., Kredel L.I., Kühl A.A., Erben U., May C., Schulzke J.D., Siegmund B. (2015). Monocyte and M1 Macrophage-induced Barrier Defect Contributes to Chronic Intestinal Inflammation in IBD. Inflamm. Bowel Dis..

[83] Vaz da Silva Z.E., Lehr H.A., Velin D. (2014). In vitro and in vivo repair activities of undifferentiated and classically and alternatively activated macrophages. Pathobiology.

[84] Arranz A., Doxaki C., Vergadi E., Martinez de la Torre Y., Vaporidi K., Lagoudaki E.D., Ieronymaki E., Androulidaki A., Venihaki M., Margioris A.N. (2012). Akt1 and Akt2 protein kinases differentially contribute to macrophage polarization. Proc. Natl. Acad. Sci. USA.

[85] Chassaing B., Aitken J.D., Malleshappa M., Vijay-Kumar M. (2014). Dextran sulfate sodium (DSS)-induced colitis in mice. Curr. Protoc. Immunol..

[86] Haneklaus M., Gerlic M., Kurowska-Stolarska M., Rainey A.A., Pich D., McInnes I.B., Hammerschmidt W., O’Neill L.A., Masters S.L. (2012). Cutting edge: miR-223 and EBV miR-BART15 regulate the NLRP3 inflammasome and IL-1β production. J. Immunol..

[87] Zaidi D., Wine E. (2018). Regulation of Nuclear Factor Kappa-Light-Chain-Enhancer of Activated B Cells (NF-κβ) in Inflammatory Bowel Diseases. Front. Pediatr..

[88] Benary U., Wolf J. (2019). Controlling Nuclear NF-κB Dynamics by β-TrCP-Insights from a Computational Model. Biomedicines.

[89] Lee J.Y., Kim J.S., Kim J.M., Kim N., Jung H.C., Song I.S. (2007). Simvastatin inhibits NF-kappaB signaling in intestinal epithelial cells and ameliorates acute murine colitis. Int. Immunopharmacol..

[90] Zhang H., Cao N., Yang Z., Fang X., Yang X., Li H., Hong Z., Ji Z. (2020). Bilobalide Alleviated Dextran Sulfate Sodium-Induced Experimental Colitis by Inhibiting M1 Macrophage Polarization Through the NF-κB Signaling Pathway. Front. Pharmacol..

[91] Zhou J., Tan L., Xie J., Lai Z., Huang Y., Qu C., Luo D., Lin Z., Huang P., Su Z. (2017). Characterization of brusatol self-microemulsifying drug delivery system and its therapeutic effect against dextran sodium sulfate-induced ulcerative colitis in mice. Drug Deliv..

[92] Zhang H., Xia X., Han F., Jiang Q., Rong Y., Song D., Wang Y. (2015). Cathelicidin-BF, a Novel Antimicrobial Peptide from *Bungarus fasciatus*, Attenuates Disease in a Dextran Sulfate Sodium Model of Colitis. Mol. Pharm..

[93] Li M., Ge Q., Du H., Jiang P., Bao Z., Chen D., Lin S. (2021). Potential Mechanisms Mediating the Protective Effects of *Tricholoma matsutake*-Derived Peptides in Mitigating DSS-Induced Colitis. J. Agric. Food Chem..

[94] Wang F., Chen Y., Itagaki K., Zhu B., Lin Y., Song H., Wang L., Xiong L., Weng Z., Shen X. (2023). Wheat Germ-Derived Peptide Alleviates Dextran Sulfate Sodium-Induced Colitis in Mice. J. Agric. Food Chem..

[95] Qi Y., Wang X., Zhang Y., Leng Y., Liu X., Wang X., Wu D., Wang J., Min W. (2023). Walnut-Derived Peptide Improves Cognitive Impairment in Colitis Mice Induced by Dextran Sodium Sulfate via the Microbiota-Gut-Brain Axis (MGBA). J. Agric. Food Chem..

[96] Paglialunga M., Flamini S., Contini R., Febo M., Ricci E., Ronchetti S., Bereshchenko O., Migliorati G., Riccardi C., Bruscoli S. (2023). Anti-Inflammatory Effects of Synthetic Peptides Based on Glucocorticoid-Induced Leucine Zipper (GILZ) Protein for the Treatment of Inflammatory Bowel Diseases (IBDs). Cells.

[97] Zhang L., Wei X., Zhang R., Petitte J.N., Si D., Li Z., Cheng J., Du M. (2019). Design and Development of a Novel Peptide for Treating Intestinal Inflammation. Front. Immunol..

