# Supplementary material for: Chensinin-1b Alleviates DSS-Induced Inflammatory Bowel Disease by Inducing Macrophage Switching from the M1 to the M2 Phenotype
Source: Biomedicines. 2024 Feb 1;12(2):345. doi: 10.3390/biomedicines12020345 (PMC10886634; doi:10.3390/biomedicines12020345)
Supplement: Supplementary file 1 [file biomedicines-12-00345-s001.zip › Figure S2.pdf]

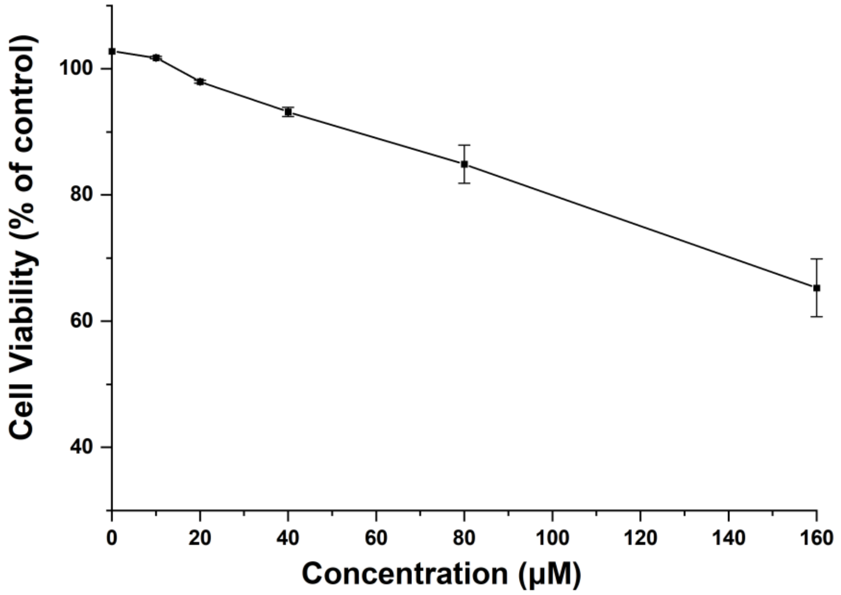

Figure S2. Effect of chensinin-1b on RAW 264.7 cell viability. Cells were administrated with gradient concentrations (0, 10, 20, 40, 80, 160 μM) of chensinin-1b for 24 h. Cell viability was test using CCK-8 assay. Data represent the mean ± SEM, n = 3.
