# Supplementary material for: Chensinin-1b Alleviates DSS-Induced Inflammatory Bowel Disease by Inducing Macrophage Switching from the M1 to the M2 Phenotype
Source: Biomedicines. 2024 Feb 1;12(2):345. doi: 10.3390/biomedicines12020345 (PMC10886634; doi:10.3390/biomedicines12020345)
Supplement: Supplementary file 1 [file biomedicines-12-00345-s001.zip › Table S1.pdf]

Table S1. Primers for qPCR

Gene Name	Sequence
Mouse GAPDH	F:5'-CGATGCCCCCATGTTTGTGA-3'
	R:5'-GAGCCCTTCCACAATGCCAA-3'
Mouse TNF-α	F:5'-TTGACCGCTTCCTCTGTACC-3'
	R:5'-TCTGGGTGTTGGAGGCTATC-3'
Mouse IL-6	F:5'-TGCTGACCTCTGGACGCTTACTC-3'
	R:5'-TCCTTAGCCACTCCTTCTGTGACTC-3'
Mouse TGF-β1	F:5'-CTCCCGTGGCTTCTAGTGC-3'
	R:5'-GCCTTAGTTTGGACAGGATCTG-3'
Mouse IL-10	F:5'-CTTAGAGCCACCCAACAAATAC-3'
	R:5'-AGAGACAGATGAGCAAGAGAC-3'
Mouse Arg1	F:5'-GACCACAGTCTGGCAGTTGG-3'
	R:5'-CACAGGTTGCCCATGCAGAT-3'
Mouse Fizz1	F:5'-GAACGCGCAATGCTCCTTTG-3'
	R:5'-CCAGTGACAACCATCCCAGC-3'
Mouse Chil3	F:5'-TACTCACTTCCACAGGAGCAGG-3'
	R:5'-CTCCAGTGTAGCCATCCTTAGG-3'
