# Supplementary figures and images for: Chensinin-1b Alleviates DSS-Induced Inflammatory Bowel Disease by Inducing Macrophage Switching from the M1 to the M2 Phenotype
Source: Biomedicines. 2024 Feb 1;12(2):345. doi: 10.3390/biomedicines12020345 (PMC10886634; doi:10.3390/biomedicines12020345)

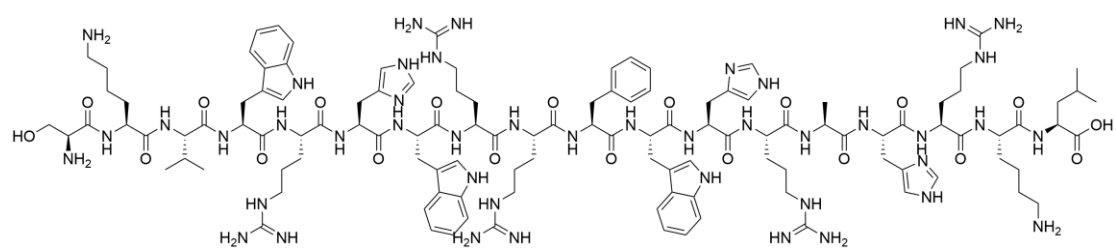

**Figure S1.** The chemical structure of chensinin-1b.

Supplement: Supplementary file 1 [file biomedicines-12-00345-s001.zip › Figure S1.pdf]
